# Perspectives on the Role of Histone Modification in Breast Cancer Progression and the Advanced Technological Tools to Study Epigenetic Determinants of Metastasis

**DOI:** 10.3389/fgene.2020.603552

**Published:** 2020-10-29

**Authors:** Jialang Zhuang, Qin Huo, Fan Yang, Ni Xie

**Affiliations:** ^1^Biobank, Shenzhen Second People’s Hospital, First Affiliated Hospital of Shenzhen University, Shenzhen, China; ^2^Shenzhen Institute of Advanced Technology, Chinese Academy of Sciences, Shenzhen, China

**Keywords:** histone modification, breast cancer, epigenetic, microfluics, next-generation sequencing – NGS

## Abstract

Metastasis is a complex process that involved in various genetic and epigenetic alterations during the progression of breast cancer. Recent evidences have indicated that the mutation in the genome sequence may not be the key factor for increasing metastatic potential. Epigenetic changes were revealed to be important for metastatic phenotypes transition with the development in understanding the epigenetic basis of breast cancer. Herein, we aim to present the potential epigenetic drivers that induce dysregulation of genes related to breast tumor growth and metastasis, with a particular focus on histone modification including histone acetylation and methylation. The pervasive role of major histone modification enzymes in cancer metastasis such as histone acetyltransferases (HAT), histone deacetylases (HDACs), DNA methyltransferases (DNMTs), and so on are demonstrated and further discussed. In addition, we summarize the recent advances of next-generation sequencing technologies and microfluidic-based devices for enhancing the study of epigenomic landscapes of breast cancer. This feature also introduces several important biotechnologists for identifying robust epigenetic biomarkers and enabling the translation of epigenetic analyses to the clinic. In summary, a comprehensive understanding of epigenetic determinants in metastasis will offer new insights of breast cancer progression and can be achieved in the near future with the development of innovative epigenomic mapping tools.

## Background

Breast cancer is the most common cancer in women, impacting 2.1 million women each year based on the data from WHO. Furthermore, breast cancer also causes the greatest number of cancer-related deaths (Fahad Ullah, 2019). It is estimated that 90% of breast cancer deaths were associated with metastasis, which is a hallmark capacity of cancer cells to spread to new areas of the body from the primary tumor. Although approximately 20–30% of early stage breast cancer will later develop to metastatic breast cancer, only a few women are diagnosed with metastatic breast cancer at the time of initial diagnosis (Peart, 2017). Breast cancer metastasis was previously considered as a complex multistep process that involves the transformation of tumor cells, changes in the microenvironment and other factors. Despite efforts that have been taken to evaluate the process of metastasis in recent years, it remains to be difficult for the diagnosis of metastasis in early stage breast cancer (Pegram et al., 2018; Scimeca et al., 2019). Breast cancer is a collection of heterogeneous diseases that differ in molecular and phenotype, which can be divided into luminal A, luminal B, HER2- enriched, and basal-like subtypes based on the expression of hormone receptors including estrogen receptor (ER), progesterone receptor (PR), and human epidermal growth factor receptor 2 (HER2). Distinct metastatic potential is found in these different subtypes of breast cancer, which resulted in different tumor progression. Therefore, a clearer basis for cancer diagnosis is necessary to identify breast cancer therapy.

For a long time, breast cancer is considered to be a disease of the genome, predominantly resulting from mutations in key genes such as *BRCA1* and *BRCA2* (Vallard et al., 2019). However, epigenetic changes mostly are demonstrated to be blamed for the cause of breast cancer progression based on the literature in the last two decades (Shukla et al., 2019). In addition to the mutations in the tumor-suppressor genes or the proto-oncogenes induced breast cancer progression, epigenetic aberrations also increase the events of breast metastasis (Chatterjee et al., 2018; Zucchetti et al., 2019). The accumulation of epigenetic changes in the tumor-related genes may contribute to the “cancer epigenome” in the alteration of gene expression without changes in the DNA sequence. In general, it is the result of a dynamic imbalance of gene expression in the body. The abnormal transcriptional regulation causes the aberrant expression of genes involved in the process of cell differentiation, survival, migration and invasion. Moreover, the epigenetic alteration of the chromosome in breast cancer cells might lead to the transition of the cell-state from the normal one to a more malignant state. The loss of the dynamic equilibrium of gene expression by epigenetic changes would contribute to the tumor progression, which might result in the metastasis at last. The current well-investigated epigenetic changes commonly involved in DNA methylation and histone modification at promoter regions of target genes, thereby increasing or reducing the expression of the genes.

## Epigenetic Drivers of Breast Cancer Metastasis

**FIGURE 1:**
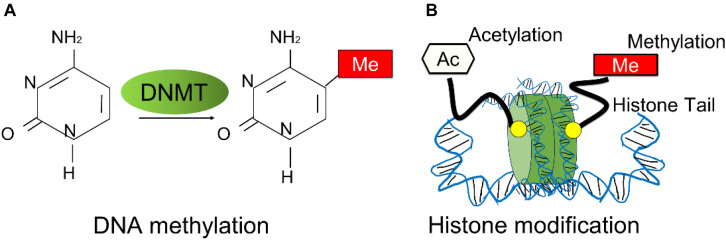
Overview of breast cancer related-epigenetic modifications. **(A)** The schematic process of DNA methylation. DNA methylation is mediated by DNA methyltransferases (DNMTs). **(B)** The schematic representation of histone modifications mostly occurred in breast cancer progression, such as histone methylation and histone acetylation.

DNA methylation is defined as the covalent bonding of a methyl group to the cytosine of the genomic CpG dinucleotide by two types of DNA methyltransferases (DNMTs), which causes changes in chromatin structure, DNA conformation, DNA stability and the way in which DNA interacts with proteins, thereby controlling gene expression (Figure 1A). DNA methylation usually inhibits gene expression, and demethylation induces gene reactivation and expression, which realizes the regulation of gene expression without changing the gene sequence. Almost all cancers have abnormal DNA methylation levels in their cells (Ehrlich, 2009). Breast cancer cells exhibit global hypomethylation and focal (gene-specific) hypermethylation which is similar to other cancers (Rauscher et al., 2015; Song et al., 2015; Benevolenskaya et al., 2016; Kanakkanthara et al., 2019). It is worth noting that DNA methylation is reversible, therefore, different from the gene suppression induced by hypermethylation of the target gene, demethylation can activate the expression of the target gene, especially tumor suppressor genes, which can also regulate tumor progression (Deshmukh et al., 2019). Although genetic changes are irreversible in principle, the DNA methylation of the target gene is dynamic, which results in the cells with the same gene achieve different phenotypes during the development. Extensive evidence has proved that cells will be undergoing a phenotypic change with DNA methylation during metastasis (Wang S.M. et al., 2019; Wu et al., 2019), which indicates such epigenetic drivers in the metastatic cascade. We speculate that specific epigenetic changes can play a key role in the process of tumor progression. The identification and study of the drivers from epigenetic change are of great significance in future breast cancer metastasis research.

Another key driver in epigenetics is histone modification, which modulates the structure of the chromatin, thereby altering the accessibility of DNA. And the main histone modifications including enzymes such as histone acetyltransferases (HATs), histone deacetylases (HDACs) and histone methyltransferases (HMTs). The acetyl groups or the methyl groups are added to the amino acid tails of the histone proteins when most of the histone modifications occur (Figure 1B). Generally, H3K5, H3K8, H3K9, H3K12, H3K18, and H4K16 refer to histone acetylation at lysine (K) of histone. For H3K4me3, the tri-methylation was added to the fourth lysine on the tail of histone. And other histone modification marks include H3K9, H3K20 and H4K27, which mono-, di, or tri-methylations at lysines of the histone proteins. Histone modification with different positions and types can both cause different effects in gene expression including activation or repression. Currently, the aberrations of histone modification in cancer can be tumor-type specific, even in the same cancer type. Breast cancer cells with different phenotypes exhibit various patterns of histone modifications, for example, lost chromatin marks-H3K27me3 was identified in a subpopulation of untreated drug-sensitive breast cancer cells (Grosselin et al., 2019). Furthermore, histone acetylation plays an important role in the development of breast cancer. HAT can significantly increase the expression of the Catechol-O-Methyltransferase (COMT) gene, which is a risk factor for breast cancer, and inhibit the survival of MCF-7 breast cancer cells stimulated by estrogen. Therefore, HDAC and HAT inhibitors can be novel options for cancer therapy, and most of drugs for breast cancer were found to be related to the alteration of the histone modification, especially for HDACs.

As we know, DNA methylation and histone modification influence each other during nucleosome remodeling and gene expression regulation, which might impact the development of the cellular processes (Du et al., 2015). For example, DNA methylation alone could not maintain a stable gene silencing directly, thus histone modifications could help to direct DNA methylation patterns and provides long-term stability of gene repression (Dumitrescu, 2012). In contrast, histone methylation only can cause reversible gene suppression, then DNA DNA methylation may be a secondary event that leads to stable long-term repression (Cedar and Bergman, 2009). Early studies mainly found that the combination of HDACis and demethylating agents or physical therapy can cause DNA methylation and histone modification at the same time, thus discovering the synergic action in TNBC or ER-α-negative breast cancer cells (Dumitrescu, 2012; Demyanenko et al., 2014; Sengupta et al., 2016; Ward et al., 2016). The subsequent studies of the underlying mechanism found that there are some interactions between the DNA methylation and histone modification, such as MAML2, UHRF1, mdig, and EZH2, which directly or indirectly induce the silencing of tumor suppressor genes in breast cancer cells (Lubecka et al., 2016; Liu X. et al., 2018; Patnaik et al., 2018; Thakur et al., 2018). In general, the inactivation of genes that occurs in these studies is a result of further DNA methylation in the genome.

In addition, another epigenetic regulatory mechanism is miRNAs, which was found to play an important role in tumor development in breast cancer. The main function of miRNA is to induce the degradation of targeted mRNA or inhibit the translation of targeted mRNA. These miRNAs can generally inhibit the expression of multiple genes and participate in the regulation of cell proliferation and differentiation. During the progression of breast cancer, the regulation of miRNA expression will lead to an imbalance in the cellular level of miRNA, and ultimately worsen the disease (Melo et al., 2014; Li Y. et al., 2015; Zhu et al., 2015; Liu et al., 2016; Ohtsuka et al., 2016; Thammaiah and Jayaram, 2016). Importantly, specific miRNAs have been found in the blood, which could be a potential biomarker for cancer (Andersen and Tost, 2020; Nassar et al., 2020). At present, a large amount of work has attempted to analyze and identify miRNA expression in different disease stages to explore the significance of these biomarkers and better clarify the potential mechanism of tumors. It is foreseeable that more miRNAs will be discovered.as a biomarker of tumors in the future, it will lead to improvements in early detection and treatment of tumors, especially in young patients.

As mentioned above, a large amount of literature has shown that epigenetics is one of the main players in breast cancer metastasis. Therefore, it is currently important to identify the role of these epigenetics in tumor metastasis and how to better analyze these epigenetic drivers related to tumor metastasis. We mainly focus on histone modification events as histone modification contributes to cancer metastasis by controlling the transition of different metastatic phenotypes in breast cancer cells.

## Role of the Histone Modifications in Breast Cancer Metastasis

**FIGURE 2:**
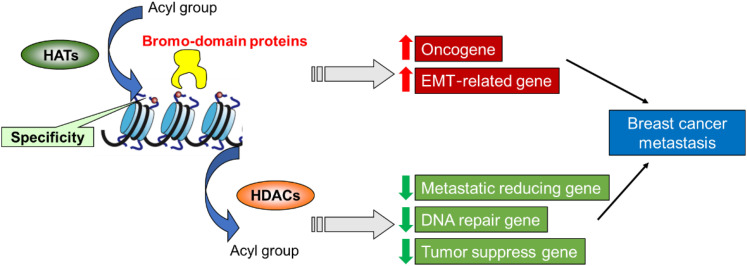
A proposed role of histone modification in the transcriptional regulation of genes involved in breast cancer metastasis and assign functional significance to these epigenetic drivers.

The histone modifications are proposed to constitute a “histone code” to maintain histone interactions with chromatin-associated proteins and therefore allow the regulation of specific downstream function. Accordingly, HATs as the “writer” enzymes could transfer acetyl groups to the particular groups of lysine or arginine residues in histone tails, which results in gene activation. In contrast, HDAC as the “eraser” enzymes could remove the acetyl groups from the tail of the histones and repress the target gene. Both “writer” and “eraser” enzymes modify histones could control the active or silent states of chromatin, which can transcriptionally regulate the transcription of genetic information encoded in DNA. Thus researchers use this to develop drugs for clinical diagnosis and treatment and provide corresponding therapeutic targets. These drugs can effectively block breast cancer metastasis and tumor progression via the impact on histone modification, especially for histone acetylation (Figure 2).

### Insight Into the Histone Acetylation

From a theoretical point of view, histone acetylation will reduce the positive charge of histones, thereby reducing the binding of nucleosomes and DNA, activating gene expression. Generally speaking, the degree of histone acetylation will be higher in the promoter region of active genes, which can affect the initiation of gene transcription and prolong gene transcription. Furthermore, histone acetylation adjusts the structure of chromatin, which in turn changes the transcriptional activity of genes. For example, the structure of acetylated chromatin becomes loose, which is related to activated transcription. The deacetylated chromatin becomes concentrated and supercoiled and is associated with transcriptional inhibition (in the case of breast cancer, it inhibits the expression of tumor suppressor genes). The acetylation reaction of histones is controlled by HATs and HADC. The reaction is a fast and reversible process. HATs are subdivided into four classes: GNAT, p300/CBP, MYST, and fungal Rtt109 family based on the catalytic mechanisms and sequence homology (Voss and Thomas, 2018). GCN5, which is from the GNAT family, was found to play a critical role in the TGF-β/Smad signaling pathway in breast cancer cells (Zhao et al., 2018). GCN5 knockdown inhibited the EMT in breast cancer and decreased the migration and invasion of MDA-MB-231 cells. Furthermore, PCAF, which is also from the GNAT family, exhibited the regulation of EMT and promotes cancer metastasis (Wang et al., 2020). p300 was demonstrated to bind with DOT1L-c-Myc complex to epigenetically induce epithelial-mesenchymal transition (EMT) regulators in breast cancer progression, which indicated that p300 served as a potential oncogene for the acquisition of aggressive phenotype of breast cancer by promoting EMT and enhances the transformation of CSCs (Cho et al., 2015). Consistent with this, Tip60 (KAT5), which belongs to the MYST acetyltransferase family, was recently reported to contribute to a reduction of H3K4 acetylation in breast cancer and the decrease of Tip60 expression could promote the tumorigenesis of ER-negative tumors (Judes et al., 2017, 2018). Most of the HATs are partially regulated by binding domains, binding to a binding partner or post-translational modifications (mainly including acetylation). Recently, histone acetyl transferase inhibitor (HATi), which can partly block the activity of the HAT, displays the potential to inhibit breast cancer growth (Shi et al., 2014). Andrographolide, a natural product which widely used in Chinese medicine could decrease the expression of COX-2 via inhibit p300 HAT activity and suppress angiogenesis through the VEGF pathway (Peng et al., 2018).

Another major category related to histone acetylation is HDACs. HDACs can be divided into 4 groups: class I (HDACs 1, 2, 3, and 8), class II (HDACs 4, 5, 6, 7, 9, and 10), class III (SIRT1, SIRT2, SIRT3, SIRT4, SIRT5, SIRT6, and SIRT7), and class IV (HDAC11) according to their sequence homology, subcellular location, and the features of the catalytic site (Chen et al., 2015). A previous study has suggested that HDAC1 can induce the growth and migration of breast cancer cells through the upregulation of Snail/IL-8 signals (Tang Z. et al., 2017). Furthermore, the CSC phenotype was well-maintained when breast cancer cells were overexpressed with HDAC1 and HDAC7, indicated that HDACs play essential roles in the CSC transition (Witt et al., 2017). HDAC2 was recently found to be critical to increase the motility of breast cancer cells via induction of metastatic markers, including MMP2 and N-cadherin (Kamarulzaman et al., 2017). The close relationship between HDAC3, EGFR and c-Src was demonstrated to promote the invasion of breast cancer cells (Kwak et al., 2019). Both HDAC3 and HDAC6 could increase the expression of survivin and siRNA treatment of HDAC3 and HDAC6 showed a similar effect of SAHA and induced autophagy in MCF7 and MDA-MB-231 cancer cells (Lee et al., 2016). miRNA-22, miRNA-10b and miRNA-125a-5p was suggested to interact with HDAC4 and contributed an impact on the drug-resistance in breast cancer cells (Ahmad et al., 2015; Hsieh et al., 2015a; Wang et al., 2018). Similar to HDAC4, HDAC5 also displays a regulatory effect on tumor resistance in breast cancer, which is related to its regulation of cell proliferation, cell differentiation and autophagy (Hsieh et al., 2015b; Li et al., 2016; Cao et al., 2017; Huang et al., 2017; Cao et al., 2018; Xue et al., 2019). HDAC8 was demonstrated to form complex with SMAD3/4 and transcriptionally suppresses SIRT7 thereby reduce metastasis and increase chemotherapy efficacy in breast cancer (Tang et al., 2020). Meanwhile, the expression of HDAC8 is related to the pathological results in triple-negative breast cancer (TNBC), which indicated the potential in the diagnosis of TNBC tumors (Menbari et al., 2020). HDAC class III sirtuins are found to be overexpressed in breast cancer. Recently, SIRT1/ERK/FOXM1 axis is demonstrated to be a critical pathway for linking metabolism to invasion and metastasis in breast cancer (Ferrer et al., 2017). Besides, the Akt pathway is also associated with the activity of SIRT1 in promoting breast cancer progression (Jin et al., 2018). And clinical analysis revealed that the impact of SIRT2 on breast cancer depends on the tumor grade, for example, SIRT 2 acts as a tumor suppressor in Grade 2 tumors while acts as a tumor promoter with Grade 3 tumor (McGlynn et al., 2014). However, the upregulation of SIRT3 could reduce migration and block metastasis in breast cancer cells (Lee et al., 2018). The expression of SIRT3 is correlated with metastatic potential in breast cancer via its control of Src/FAK signaling. Both FOXO6 and RUNX2 could suppress the expression of SIRT6, which caused the reduction of cell migration, invasion, and proliferation of breast cancer cells (Choe et al., 2015; Ye and Duan, 2018). Our recent works have demonstrated that SIRT7 reduced breast cancer metastasis via inhibiting the TGF-β signaling (Tang X. et al., 2017). The results found that SIRT7 promoted the degradation of SMAD4, which is the key factor in TGF-β pathway, which indicated the potential of the therapeutic target for SMAD4-mediated breast cancer. Furthermore, SIRT7 is also suggested to serve as a prognostic biomarker in breast cancer based on previous studies (Aljada et al., 2015; Huo et al., 2020). It is worth noting that HDACs have become important therapeutic targets in various cancer with the increasing number of reports on the HDAC inhibitors (HDACi). There are currently 5 HDAC drugs on the market (Zucchetti et al., 2019). Four HDAC inhibitors were approved by the US FDA for the clinical treatment of peripheral T-cell lymphoma, cutaneous T-cell lymphoma and multiple myeloma, and one was approved by the China Food and Drug Administration for peripheral T-cell lymphoma. Furthermore, a number of HDACi were investigated in the treatment of solid tumors, including breast cancer (Huang et al., 2019; Ediriweera and Cho, 2020). HDACi could prevent breast tumor progression via transcriptional reduction of EMT, induction of ER in hormone receptor-negative tumors, improving the sensitivity of hormonal therapy in ER + tumor, or modulating the expression of HER2 (Connolly et al., 2017). So far, HDACi is the first successful epigenetics-based anti-tumor drug, especially against hematological malignancies. At present, only one phase III clinical trial proves that HDACi exhibits anti-tumor effects in breast cancer (Yeruva et al., 2018), but it has not been approved for clinical use, but it can become a promising class of drugs for the treatment of all breast cancer subtypes, especially for the refractory hormone-positive disease. At the same time, through the combination of immunotherapy, HDACi can also have a certain inhibitory effect on metastatic TNBC (Terranova-Barberio et al., 2017).

### Insight Into the Histone Methylation

Histone methylation is a covalent modification that occurs on arginine and lysine. Arginine can be monomethylated or dimethylated, and lysine can be monomethylated, dimethylated or trimethylated. Similar to DNA methylation, the process of histone methylation involves the transfer of methyl groups from S-adenosylmethionine (SAM) to lysine or arginine residues by HMT, and histone demethylase (HDM) removes methyl groups from the histone. The expression of HMTs might impact on the progression of tumor and metastasis. For example, PRMT1, which is a targeted HMT, could bind to the promoter of ZEB1 and mediates histone methylation to induce the EMT process in breast cancer cells (Li et al., 2017). Furthermore, H3K9me3, which refers to the trimethylation of histone third subunit No. 4 lysine, was associated with the metastasis of aggressive breast cancer based on the global DNA methylation analysis (Thakur et al., 2018). An additional study found that PRMT2 could improve the sensitivity of tamoxifen in ER + breast cancer cells through transcriptional suppression of ER-α36 (Shen et al., 2018). Activation of PRMT4 was suggested to inhibit endocrine-resistant breast cancer via an epigenetic mechanism (Hiken et al., 2017). PRMT5 is the most promising target for the treatment of breast cancer based on the principle of histone methylation. A clinical study has suggested that PRMT5 is associated with poorer clinical outcomes. In concordance, PRMT5 is reported to bind to the promoter of FOXP1 and enhance the number of CSC in breast cancer (Chiang et al., 2017). Further analysis found that the inhibition of PRMT5 in breast CSC could lead to the reduction of metastatic potential and lower proliferation of the cells (Chiang and Davies, 2018). Moreover, PRMT5 was also reported to regulate drug sensitivity in breast cancer cells (Chen et al., 2017). To summary, PRMT5 is related to poor clinical outcome and the transition of CSC in breast cancer, which provide a potential target for inhibition of breast cancer metastasis. PRMT6 is associated with increased recurrence and promoted metastasis of breast cancer via the attenuation of p21 (Nakakido et al., 2015). Similar with PRMT5, PRMT7 could promote the EMT process and increase cell migration in breast cancer cells. Further analysis found that the regulation of PRMT7 is directly mediated by the increase of E-cadherin (Geng et al., 2017). However, the expression of PRMT8 is clinically related to improved breast cancer survival in the impact on breast cancer proliferation (Hernandez et al., 2017). And PRMT9 could promote cell migration and invasion via the EMT process and increased PI3K/Akt/GSK-3β/Snail pathway (Jiang et al., 2018). Although HMTs are reported to be correlated with the progression of breast cancer while HMT inhibitors targeting PRMT3, PRMT4, PRMT5, and PRMT6 have been reported to display tumor inhibition preclinically, few drugs can progress to the clinical stage (Wang S.M. et al., 2019). However, the results of early clinical trials of PRMT5 are still worthy of attention (Li et al., 2019), that is, this target has great prospects and possibilities in the treatment of breast cancer.

### Insight Into Other Histone Modifications

The phosphorylation modification of histones H2B and H3 was demonstrated to play an important role in DNA repair, mitosis and gene regulation (Magerl et al., 2010). A previous study has proved that there are abnormal histone phosphorylation in the histone modification profile of breast cancer cells, which might be related to the expression of Jumonji domain-containing 6 (JMJD6; Liu et al., 2019). Furthermore, H2AX phosphorylation (γ-H2AX) was found to inhibit histone methylation via the binding to PRMT1 in cancer patients with overexpression of protein phosphatase 2A (PP2Ac), thereby increasing the level of histone phosphorylation (Thompson et al., 2013). Therefore, the overexpression of histone phosphorylation in cancer cells was regarded as a biomarker of cancer in recent years (Duong et al., 2010). In contrast, the ubiquitination of histones H2A and H2B in breast cancer cells mainly plays an important role in gene transcription and maintaining genome integrity (Cole et al., 2015; Janaki Ramaiah and Vaishnave, 2018). For instance, BMI-1 (B-lymphoma Mo-MLV insertion region 1)which is a key player in the ubiquitination of histone H2A, was found to be correlated with advanced stages of breast cancer. The H2Aub could affect gene expression involved in EMT and cancer stemness in cancer cells (Sethi et al., 2018). And BRCA1-related protein 1 (BAP1) was observed to catalyze the removal of ubiquitin groups of H2A, which might be involved in the development of breast cancer (Jiao et al., 2013). Sumoylation of histones is a reversible post-translation modification that entails covalent addition of small ubiquitin-like modifier (SUMO) to histone proteins (Fu et al., 2019). Sumoylation is commonly regards as a negative regulation and suppress the transcripitional activity of the target gene. Increasing evidence suggest that sumoylation is known to regulate histone modifying enzymes including HDAC1, HDAC2, HDAC4, SIRT1, EZH2, and KDM5 (Shanmugam et al., 2018). Furthermore, a recent study claimed that there is a cross-talk between chromatin acetylation and histone SUMOylation, which could impact the cell adhesion in breast cancer cells (Appikonda et al., 2018). Histones are known targets for ADP-ribosylation. Histone ADP-ribosylation is a reversible histone modification by ADP-ribose polymerases (PARPs), resulting from the transfer of an ADP-ribose moiety from NAD^+^ to specific residues (Hu et al., 2019). The ADP-ribosylation is demonstrated to maintain active transcription, which allows an open chromatin structure. It is generally related to cell proliferation and DNA repair in breast cancer cells. PARP1, which is the most study nuclear ADP-ribosyltransferases, is found to account for up to 90% of the total cellular poly ADP-ribosylation and can target all five histone proteins (Jain and Patel, 2019). Recent works have also indicated that there is an overexpression of PARP1 in human cancer tissue when compared with the adjacent non-tumor tissues (Na et al., 2016). And PARP1 is also demonstrated to bind with other transcription promoters during DNA repair in breast cancer (Hu et al., 2014; Sobczak et al., 2019).

### Insight Into the Subtype-Specific Associations Between Histone Modifications and Breast Cancer Metastasis

As we know, the classification of breast tumors is based on their hormone receptor status and pathologic features and the histone modifications play important roles in the regulation of gene expression in cancer pathogenesis. Thus there might be a subtype-specific regulation in breast cancer which is related to histone modifications. Recently, efforts have been taken to investigate the association between histone modifications and the subtypes of metastatic breast cancer (Li D. et al., 2015; Lee et al., 2017; Xi et al., 2018). For example, histone acetylation H3K9ac was observed to be correlated with HER2-positive and TNBC subtype while histone methylation H3K27me3 was comprised of Luminal A and B1 subtypes (Karsli-Ceppioglu et al., 2017). Another nurses’ health study confirmed that H3K27me3 was associated with lower grade tumors and the luminal A subtype (Healey et al., 2014). Moreover, the epigenetic modifier Ezh2 was found to maintain H3K27me3-mediated repression of the FOXC1 gene in Luminal B breast cancer specifically, resulting in the process of metastatic behavior in both a mouse model and patient-derived xenografts (Hirukawa et al., 2018). A ChIP-seq study indicated that H3K36me3 was commonly found in HER2 positive breast cancer and both H3K4me3 and H3K79me2 were overexpressed in TNBC cell lines (Xi et al., 2018). A further study has accessed the relationship between the SIRT1, H3k4ac, H3k9ac, and H4k16ac in different subtypes of breast cancer and found that SIRT1 is upregulated in luminal and HER2-enriched subtypes and significant downregulation in TNBC subtype while H3k4ac, H3k9ac, and H4k16ac were significantly reduced in luminal and HER2-enriched subtypes and relatively upregulated in TNBC subtype (Rifai et al., 2018). Meanwhile, the histone variants including H2AX, MACROH2A.1, and H2Bub1 and histone chaperones such as APLF and HJURP were identified as the potential epigenetic biomarkers targeting specific subtypes of breast cancer (Nandy et al., 2020)

## Novel Approaches to Study Epigenetic Determinants of Metastasis

With the efforts to advanced therapeutics in the treatment of breast cancer, yet breast cancer metastasis remains the leading cause of death in women patients. The major reason is that the epigenome effects in breast cancer metastasis were still unclear. Currently, available technologies allow the study the epigenetic drivers in breast cancer metastasis, however, these traditional tools limited the accuracy and precision in the investigation of epigenomic signatures, which might enlarge the gaps between promise and realization of epigenomic therapy. Therefore, concerns should be taken to develop novel approaches to better describe the epigenome in breast cancer metastasis. The following sections here purposed the current new technologies for profiling epigenetic determinants of breast cancer metastasis, including the next-generation sequencing (NGS) and microfluidic platform for mapping the epigenome recently.

### Next-Generation Sequencing in the Study of Breast Cancer Epigenomics

**FIGURE 3:**
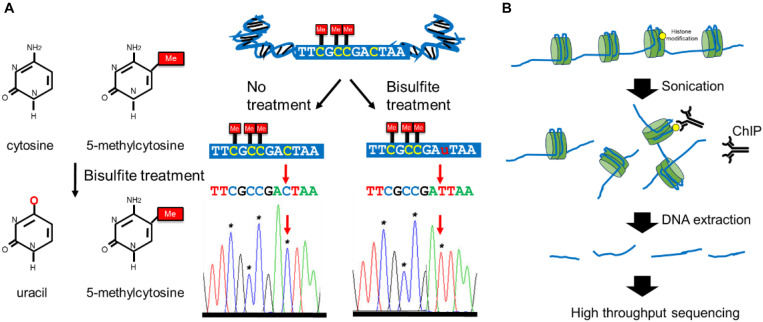
Overview of the major epigenetic modification detection methods. **(A)** The working mechanisms of bisulfite conversion-based methods for DNA methylation mapping. **(B)** The workflows of ChIP-seq methods for assessing genomic loci associated with specific histone modifications.

For many years, the research of breast cancer epigenetics has been only aimed at the DNA methylation analysis of a specific gene and the identification of histone modifications. These research results have laid the foundation for the study of breast cancer epigenetics, and revealed that the epigenetic information can be used as a new generation of clinical diagnostic markers and new targets for anti-tumor therapy. The technological development of NGS in recent years has promoted the progress in the study of breast cancer epigenetics gradually from a single gene to a genome-wide scale. Recently, whole genome bisulfite sequencing (WGBS) is the gold standard for DNA methylation research (Cokus et al., 2008). It uses a combination of Bisulfite processing and whole-genome DNA sequencing to analyze the methylation of the entire genome (Figure 3A). In addition to WGBS can be used to detect the degree of DNA methylation, there are (Reduced representation bisulfite sequencing) RRBS, oxidized-bisulfite sequencing (oxBS-Seq), TET-assisted bisulfite sequencing (TAB-seq), Methylation sensitive restriction enzyme sequencing (MRE-Seq), HELP-Seq, methylated DNA immunoprecipitation sequencing (MeDIP), methylation binding domain capture technology (MBD-CAP) and probe-based targeted enrichment technology (Meissner et al., 2005; Weber et al., 2005; Khulan et al., 2006; Rauch et al., 2008; Fouse et al., 2010; Miura et al., 2012; Yu et al., 2012; Booth et al., 2014). Due to the fact that existing DNA extraction methods do not have much effect on DNA methylation, the basic principles of the above technologies are mostly based on the reaction of sodium bisulfite treatment of DNA. However, the bisulfite-based sequence data require more complex bioinformatics analysis methods (Rauluseviciute et al., 2019). Although many excellent tools have been developed specifically designed to process bisulfite sequence data, it is still difficult for researchers who do not have sufficient experience in data analysis and bioinformatics to select the most suitable analysis tool. In addition, Illumina sequencing also faces the same problems as all short-read data, especially mapping the problem to repetitive or low-complexity regions (Gouil and Keniry, 2019). The loss of sequence diversity caused by bisulfite conversion further exacerbates these problems. The long-read sequencing method developed in recent years provides a certain solution to the above problems. There are currently two main long-read sequencing technologies: Oxford nanopore technology (ONT) nanopore sequencing and Pacific Biosciences (PacBio) single molecule real-time (SMRT) sequencing (Eisenstein, 2012; Kingan et al., 2019). The basic principle is to sequence natural DNA, so as to analyze the modification of the base through the result of the original sequencing signal. However, due to the inability to amplify DNA, the amount of input for detection is limited. The long-read sequencing method has very high requirements for samples. If you only focus on specific genes or partial regions of the genome, both PacBio and ONT can use PCR-free methods, such as CRISPR-based enrichment technology, to achieve hundreds or thousands of times of gene enrichment to increase the input of sequence, which might overcome the disadvantage of these approaches (Hafford-Tear et al., 2019; Gilpatrick et al., 2020). In general, both short-read bisulfite sequencing and long-read sequencing have their own obvious advantages and limitations, which affect their applications. In actual work, scientists need to select appropriate NGS for DNA methylation analysis based on the characteristics of samples.

Methods based on NGS are also extensively used to study histone modification in recent years. Chromatin immunoprecipitation-deep sequencing (ChIP-Seq) is a high-throughput technology that combines chromatin immunoprecipitation (ChIP) with deep sequencing technology (Visel et al., 2009; Figure 3B). This technology provides a high-resolution, low-noise, and high-coverage research method to identify where histone modifications are in the whole genome. This technology first uses specific histone modification antibodies to enrich the DNA region bound by histone modification, and then performs high-throughput sequencing on this region after purification and library construction. However, ChIP-seq requires a very large amount of sample input (10^6^–10^7^) and is difficult to apply to the analysis of rare samples. The recently developed ULI-NChip technology has solved this problem and determined the H3K4me3 and H327me3 histone modification regions on the order of 10^3^ cells for the first time and the low-input Chip-seq histone modification analysis has been successfully achieved (Brind’Amour et al., 2015; Harada et al., 2019). Mass spectrometry (MS) has been used to characterize histone modification (Karch et al., 2013). This involves the extraction and purification of histones by acid extraction or high salt extraction. Then middle-down MS or top-down MS was used to analyze purified protein, which can detect the modified histones with enzymatic hydrolysis (middle-down approach) or without enzymatic hydrolysis (top-down approach; Chakravarthi et al., 2016). High-resolution analyzers (such as Orbitrap) are often used for histone modification analysis because they can distinguish histone modification with almost the same mass signatures (for example, histone acetylation at 42.0106 Da and histone tri-methylation at 42.0470 Da; Karch et al., 2014). However, for low input histone modification analysis, high-sensitivity instruments are still needed to obtain more accurate signals. At present, the improvement of mass spectrometry-based analysis methods for histone modification is mostly focused on histone pretreatment methods, such as improving the integrity of histones after extraction, increasing the amount of extracted histone, and improving the purity of separation. Due to the characteristics of rapid enrichment, sample consumed in the nanoliter range, high separation efficiency and online separation and detection, coupling capillary electrophoresis (CE) to mass spectrometry has become one of the best methods for histone modification analysis in recent years (Faserl et al., 2020).

Deep RNA sequencing (RNA-seq) has been used to reveal the roles miRNAs play in epigenetic regulation in recent years. Among many NGS technologies, the first method used for miRNA detection is the second-generation methods including Roche’s 454 pyrosequencing and Illumina (Solexa) sequencing (Liu et al., 2020). The sequencing steps of these two methods are basically the same, including sample preparation, template preparation, sequencing, and imaging, small RNA reads assembly, alignment and so on. Among them, Roche’s 454 pyrosequencing is based on a PCR reaction in a microemulsion, while Solexa Sequencing technology uses bridge PCR for amplification. The general workflow starts with the RNA-cDNA library, which is formed by reverse transcriptase. After the adaptor is enzymatically connected to the two ends of the mature miRNA, reverse transcription of the small RNA fragment can be performed, and finally the sample is analyzed after amplification by massively parallel sequencing. Both of them can not only measure the expression level of miRNAs, but also detect small changes in the length and sequence of known miRNAs, which are important for mining new miRNAs. In addition to these two methods, the SOLID sequencing technology introduced by ABI was also the main sequencing platforms with the highest accuracy (99.99%) among the second-generation methods (Goodwin et al., 2016). However, this method still has problems. Firstly, the read length is relatively short, and secondly, it is difficult to guarantee false positives during the amplification process since SOLID sequencing is based on the PCR reaction. Therefore, the third- and fourth-generation sequencing methods are non-PCR-based methods, such as true single molecular sequencing (tSMS), single-molecule real-time sequencing (SMAT), fluorescence resonance energy transfer (FRET), and the United Kingdom company’s nanopore single-molecule sequencing (Technology; Ardui et al., 2018; Kumar et al., 2019). Nevertheless, considering the cost and applicability of samples, the most commonly used miRNA sequencing method is still the second-generation sequencing system.

### Microfluidic Technology to Study Breast Cancer Epigenomics

In addition to NGS, microfluidics provides efficient platforms to maintain epigenomes study on DNA methylation, histone modifications and 3D chromatin structures (Deng et al., 2019). Microfluidic assays allow precise control of small amounts of fluids with automated and high-throughput operations (Oliveira et al., 2016). In the past few decades, microfluidic technology has set off a new wave of rapid development in cell biology and molecular biology related fields such as single-cell analysis (Zhuang et al., 2018; Xu X. et al., 2020), biological tissue model construction (Sontheimer-Phelps et al., 2019; Zhuang et al., 2019), and biochemical analysis (Campbell et al., 2018; Wang Z. et al., 2019; Qin et al., 2020). Currently, efforts have been taken to use microfluidic devices as powerful approaches to process epigenomic assays (Xu Y. et al., 2020).

Bisulfite conversation-based detection is widely used in DNA methylation analysis in recent years. As a high-throughput and automated device, the microfluidic platform allows bisulfite treatment with higher efficiency when compared with traditional methods. The on-chip bisulfite conversion was processed in a microfluidic chip which was integrated with the MS-PCR analysis unit, the device has achieved the methylated DNA detection within 80 min with low-input sample (Yoon et al., 2015). These microfluidic chips with capabilities of bisulfite conversion can not only be integrated with self-made testing equipments (Li Z. et al., 2015; Heng et al., 2017), but also can be used in combination with commercial testing equipment, for example, the bisulfite conversion of DNA by a microfluidic chip was reported to be subjected to Fluidigm, which successfully accessed multiple CpG loci in multiple DNA samples with high reproducibility (Adamowicz et al., 2018). Researchers also try to use the analysis of DNA melting temperature to determine the methylation of one or more DNA regions. Among them, CE is the earliest method used (Fang et al., 2012). The CE-based microfluidics derived from CE is also following the above principle (Zhang et al., 2013), but the sensitivity of this type of method is relatively low, and the background noise is large which seriously affected by the accuracy of the detection. Therefore, droplet microfluidic technology is the current development direction of methods based on this principle, which can distinguish the unique melting temperature of each individual mixed methylation droplet, thereby realizing sequencing-free gene methylation analysis at a low cost (Liu F.W. et al., 2018). In recent years, a high-throughput analysis method, HYPER-Melt, that uses methylation-sensitive high-resolution melt (MS-HRM) to detect DNA melting temperature has also emerged (O’Keefe et al., 2018). In other words, this method extends the DREAMing [Discrimination of Rare EpiAlleles by Melt (Pisanic et al., 2015)] method to the microfluidic device for use. The DREAMing technology is as follows the bisulfite-converted DNA is first loaded into a 96-well plate, then the samples are amplified by the PCR method. The PCR amplification method will preferentially amplify methylated DNA to increase the corresponding signal. Finally, by distinguishing the signal intensity between samples, the methylated region can be detected based on the melt curves. HYPER-Melt is equivalent to amplifying the method to 4,096 microwells in a microfluidic chip for analysis to determine the signal intensity in different microwells, and finally to analyze DNA methylation. Bisulfite-free methods were recently developed to overcome the drawbacks of bisulfite conversion such as DNA degradation and long-time treatment. A microfluidic device is reported to determine methylated DNA rely on antibodies that bind to methylated DNA (Hasegawa et al., 2016). This method detects the methylated DNA by fluorescently labeled streptavidin using the affinity between the biotin-antibody and the methylated DNA. Since its principle is similar to the traditional ELISA method, the operation is simple and the experiment time is short, which is friendly for clinical application. The microfluidic chip method based on electrochemical sensors can also use this principle to measure the concentration of methylated DNA (Hong et al., 2018). A microfluidic device that performed methylated DNA immunoprecipitation (MeDIPseq) is also developed to detect methylated DNA concentration at the fM-scale (Zhu Y. et al., 2019).

Chromatin immunoprecipitation is used as the gold standard to analyze histone modifications, however, limitations of ChIP including large sample size, low throughput, and poor robustness prevent its routine implementation. Recent efforts have been taken to improve ChIP assay with microfluidic tools by reducing the reagent volume, maintaining multiple samples parallelly and process workflow automated (Wu and Quake, 2016). AutoChIP was developed to decrease the incubation time and the required sample via integrated with a microfluidic platform (Wu et al., 2012). The whole device consists of multiple flowing channels and a valve-control system, which are used to precisely manipulate the liquids in the ChIP process. Moreover, the ultrasonic sonication was also recently integrated into the microfluidic chip in order to facilitate efficient mixing and washing in the ChIP process (Cao and Lu, 2016). A droplet-based microfluidic chip was reported to reduce cross-contamination and enhanced mixing in the nanoliter scale (Teste et al., 2017). The droplet microfluidic device can also optimize the fragmentation of chromatin process to improve the efficiency of downstream epigenomic assays, such as ChIP (Xu et al., 2018). This method digested the chromatin of 2,500–125,000 cells through the automatic processing of the microfluidic chip, which demonstrated that the obtained nucleosome has higher efficiency and better purity than traditional methods. However, this method is not suitable for single-cell analysis. Recently, a droplet-based microfluidic system was developed to study the histone modification of single cells, which can maintain efficient single-cell encapsulation in the droplets, thus the separation of single cells, the pretreatment of cells, the extraction of DNA and the process of ChIP are all integrated on a chip, providing comparable signals and higher sensitivity (Kim et al., 2018). There are also microfluidic chip methods dedicated to improving the extraction efficiency of ChIP DNA for histone modifications detection. For example, MOWChIP (microfluidic oscillatory washing based ChIP) was established to collect ChIP-DNA using less than 100 cells via a fast and complete immunoprecipitation on chip (Cao et al., 2015; Cox et al., 2019; Zhu B. et al., 2019). In this study, oscillatory washing is essential for reducing non-specific binding after the ChIP process, which can increase the purity of collected ChIP-DNA. In addition, SurfaceChIP-seq, where the microchannels were coated with antibodies for immunoprecipitation, was used to capture the specific DNA from the flowing chromatin (Ma et al., 2018). There is also LIFE-ChIP (low-input fluidized-bed enabled ChIP) using programming design and solenoid valve control to further automate the ChIP process (Murphy et al., 2018).

Different from the analysis methods for DNA methylation and histone modification, miRNA detection is mainly based on RNA-sequencing technologies. However, many microfluidic chips have tried to measure miRNA in recent years without RNA-sequencing. For example, a SERS-based microfluidic device was used to detect miR-222, which is involved in multiple cancers, with silver-coated porous silicon membranes (Novara et al., 2017). The chip has integrated the biosensor in the device, which offers a label-free diagnosis approach for early cancer. The electrochemical biosensor could be also combined with a microfluidic chip. Recently, miRNA-197, which could be used as a tumor biomarker, was measured with a low-cost electrochemical microfluidic biosensor platform (Kutluk et al., 2020). Furthermore, a laser-induced fluorescence detection system also could be integrated with a microfluidic chip, allowing the determination of miR-21 and miR-31 in A549 and HeLa cells (Cai et al., 2020). Meanwhile, a microfluidic miRNA detection strategy using a gold deposition-based signal amplification scheme and dark-field imaging was developed with a limit of detection of 260 fM (Lee et al., 2020). Other microfluidic chips are based on the rolling circle amplification to improve the sensitivity of miRNA detection (Cao et al., 2019; Treerattrakoon et al., 2019; Smith et al., 2020). Although most of these methods could facilitate accurate miRNA analysis with a limited sample, however, most of them required additional nanoparticles-based sample preparation, which is hard for clinical applications. Point of care (POC) systems were also developed in recent years. A miRNA-based microfluidic POC platform integrated with fluorescence reader was demonstrated to screen and detect miR-21 in the blood samples of breast cancer patients within 30 min (Salim et al., 2017). A further step in this direction was taken by using a power-free microfluidic chip to detect miR-196a and miR-331(Kim et al., 2019). The miRNA detection of the device was based on the fluorescence signals after sandwich hybridization between probe DNAs and target miRNA. In addition to the previously mentioned microfluidic platforms without RNA-sequencing, RNA-sequencing-based commercial microfluidic devices have been used in miRNA analysis (Lazar et al., 2018; Zhou et al., 2018). For example, μParaflo from LC Science was an RNA-array-based microfluidic platform that could profile miRNA expression using highly parallel RT-PCR (Shen et al., 2013). Other microfluidic chips are involved in the TaqMan array and the Fluidigm C1 platform (Rizzo et al., 2017; Takousis, 2018), which is used to identify differential miRNA expression in cancer cells.

## Future Directions in Epigenomic Mapping for Breast Cancer Metastasis

Increasing evidence shows that epigenomic can indicate the progression of breast cancer. The epigenomic profiles including DNA methylation and histone modification and so on may lead to abnormal protein expression in signaling pathways related to tumor metastasis, and finally cause tumor progression. We demonstrated that epigenetics plays a key role in breast cancer metastasis, but it also leaves many important open questions. For example, because the tumor microenvironment also plays an important role in breast cancer metastasis, whether the tumor microenvironment is also changed by the epigenetic changes of tumor cells. Then, since EMT is involved in multiple processes of breast cancer metastasis, it is usually temporarily activated or partially activated in breast cancer cells. Which histone modification enzymes such as the SIRT family play an important role in it? What signaling pathways do these upstream regulatory genes use to affect downstream gene expression and affect the balance between EMT cell state at last? In addition, we have described that there are a variety of enzymes involved in the change of epigenetics, however, the analysis methods for proteins or enzymes are still lacking. The analysis of these enzymes including DNMT, HMT, HDAC, etc., lacks high-throughput analysis methods. Finally, is there any relationship between epigenetic instability and the heterogeneity of tumor cells? Does the reversibility of such epigenetics such as methylation and histone modification increase tumor heterogeneity and affect tumor metastasis.

By analyzing the relationship between epigenetic information and breast cancer metastasis, the epigenome mechanism of breast cancer metastasis can be clarified, so as to provide insights for diagnosis and treatment. There are many new methods including NGS and microfluidics that can be used to detect epigenetic information such as DNA methylation and histone modification, but from the current progress, the work of single-cell epigenome analysis is less than that of single-cell genome and single-cell transcriptomics. Therefore, the powerful analysis capabilities of NGS and the miniaturization and integration characteristics of microfluidic chips should be combined to promote the investigation of single-cell epigenetics of breast cancer metastasis. Secondly, in the future, epigenetics research will also develop in a direction that is more suitable for early clinical screening. For example, to reduce the number of testing samples, improve the high-throughput analysis capabilities of the device, and offer automatic process, the development of novel microfluidic chips makes the analysis of these epigenetic biomarkers as easy and simple as clinical biochemical analysis. Finally, these methods will need to be validated using clinical samples (such as tissue biopsies) and compared with batch methods to ensure accurate data coverage before they can be reliably applied in the clinic.

All in all, the progress on epigenetics in breast cancer metastasis helps to better understand the molecular mechanisms associated with metastasis, thereby helping to accelerate the development of new metastatic treatment strategies and biomarkers.

## Author Contributions

JZ and NX contributed to the conception of the manuscript. JZ wrote the manuscript. QH and FY revised the manuscript. All authors gave approval to the final version of the manuscript.

## Conflict of Interest

The authors declare that the research was conducted in the absence of any commercial or financial relationships that could be construed as a potential conflict of interest.
